# Immune suppression of IgG response against dairy proteins in major depression

**DOI:** 10.1186/s12888-017-1431-y

**Published:** 2017-07-24

**Authors:** Leszek Rudzki, Dariusz Pawlak, Krystyna Pawlak, Napoleon Waszkiewicz, Aleksandra Małus, Beata Konarzewska, Mirosława Gałęcka, Anna Bartnicka, Lucyna Ostrowska, Agata Szulc

**Affiliations:** 1 Department of Psychiatry, Medical University of Bialystok, Bialystok, Poland; 2 Department of Pharmacodynamics, Medical University of Bialystok, Bialystok, Poland; 3 Department of Monitored Pharmacotherapy, Medical University of Bialystok, Bialystok, Poland; 4 Institute of Microecology, Poznan, Poland; 5 Department of Dietetics and Clinical Nutrition, Medical University of Bialystok, Bialystok, Poland; 6 Department of Psychiatry, Medical University of Warsaw, Warsaw, Poland; 7 Argyll and Bute Hospital, Blarbuie Road, Lochgilphead, PA31 8LD Scotland UK

**Keywords:** Major depression, IgG, TNF-α, Cortisol, Leaky gut, Gut-brain axis, Food allergy, Tight junctions

## Abstract

**Background:**

Interactions between the digestive system, brain functions and immunoglobulin G (IgG) mediated immunity against food antigens became recently a topic of growing interest in psychiatry research. Psychological stress can activate hypothalamic-pituitary-adrenal axis (HPA) with subsequent hypercortisolemia. It can also influence intestinal permeability and dynamics of IgG response. Major depression can by accompanied either by activation of inflammatory response or by immune suppression (e.g. decreased antibody production) where hypercortisolemia is a significant immune modulator.

The aim of our study was to assess IgG immune response against 44 food products in depressed patients and controls along with markers of psychological stress, inflammation, psychometric and dietary parameters.

**Methods:**

Serum IgG concentrations against 44 food antigens, plasma cortisol, TNF-α, IL-6, IL-1b concentrations were measured and psychometric parameters were evaluated using Hamilton Depression Rating (HAM-D 17), Perceived Stress (PSS-10), and Symptom Checklist (SCL-90) scales in 34 depressed patients and 29 controls. Dietary parameters such as frequency of exposure to food antigens, appetite and weight change were assessed.

**Results:**

There was a significantly lower IgG concentration against dairy in depressed patients compared to controls (post hoc *p* < 0.05) when there was a high exposure (consumption) to dairy. Our research revealed a significant interaction of IgG concentration against dairy proteins and exposure to dairy between groups (F (2.63) = 3.92, *p* = 0.025, η^2^ = 0.12). There was no significant difference in mean IgG concentration against food antigens between patients and controls.

We found increased concentration of cortisol in depressed patients (t (1.61) = 2.37, *p* = 0.02) compared to controls. Patients with melancholic depression had significantly higher (*M*_*rank*_ = 21.27) concentration of cortisol (*U* = 41, *p* = 0.006), when compared with the non-melancholic group of patients (*M*_*rank*_ = 12.16). Cortisol concentration significantly positively correlated with HAM-D 17 (*r* = 0.442, *p* = 0.009) and with phobias in SCL-90 scale in patients’ group (*r* = 0.531, *p* = 0.001). There was decreased concentration of TNF-α (*t* = 4.256, *p* < 0.001) in depressed patients compared to controls. IgG concentration of 38.63% food products positively correlated with TNF-α concentration in depressed patients compared to 9.09% of those in healthy controls.

**Conclusions:**

We observed an immune suppression of IgG response to dairy proteins in depressed patients. Hypercortisolemia with involvement of decreased concentration of TNF-α might play a significant role in suppression of IgG response in depressed patients.

## Background

Through recent years, there has been a great increase in research interest in the topic of interactions between digestive system and brain functions. These bidirectional interactions take place in the so called gut-brain axis, or microbiota-gut-brain axis [1]. The intestinal barrier, with its surface of ∼300 m^2^ plays a crucial role in the interaction between the internal and external environment of the human organism. As it is the largest immune organ in the human body, its functions are critical in immunomodulation and antibody production and these functions are substantially influenced by food consumption and exposure to food antigens. Moreover, psychological stress can substantially influence its physiology and permeability [2]. Also, the coexistence of psychiatric illnesses with multiple gastrointestinal disorders e.g. inflammatory bowel disorders (IBD) and irritable bowel syndrome (IBS) indicate the significance of the gut-brain axis in the context of mental health.

Interactions between psychological stress, major depression symptomatology and immune system are immunologically multidimensional phenomena. For many years psychological stress and depression have been associated with suppression of cellular and humoral immunity and higher susceptibility to infectious and neoplastic diseases [3, 4]. Some examples of such responses to stress are decreased IgG antibody production after vaccinations [5–7], progression of AIDS in HIV infected patients [8], suppression of lymphocyte stimulated responses, their mitogenic activity and immunoglobulins production [9–11]. Moreover, it has been demonstrated that patients with major depression have a decreased number and impaired functions of natural killer (NK) cells [12–17], decreased absolute numbers of T and B cells and decreased neutrophil functions [18, 19]. Psychological stress can also lead to suppression of genes involved in interferon-mediated innate antiviral responses and immunoglobulin G production [20].

Hypercortisolemia is one of the common features of major depression and it can be identified in about 60% of depressed patients [21]. It is also a well-known effect of HPA axis activation due to psychological stress and it is believed to be one of the major factors of immune suppression. It also has a detrimental effect on B lymphocytes functions which are responsible for antibodies production. For example, chronic psychological stress among doctoral students undergoing qualifying exams was shown to dramatically decrease their levels of CD19+ lymphocytes when compared to matched community controls [22]. In this study the level of stress was assessed using the Perceived Stress Scale (PSS) and there was a significant negative correlation between CD19+ lymphocyte percentage and PSS score. Human in vitro studies revealed very high sensitivity of early B lymphocytes (CD 10+, CD19+) to glucocorticoids. Those hormones led to loss of 60–80% of early B lymphocytes due to apoptosis [23, 24]. Moreover, in mouse models, high but physiological levels of glucocorticoids were associated with 50% reduction in peripheral blood B lymphocytes [25]. Multiple mechanisms have been described showing how glucocorticoids could induce cell death through apoptosis. For example, glucocorticoids downregulate c-myc protein which is important in cell proliferation and cell survival [26]. Furthermore, those hormones downregulate cyclin dependent protein kinases required for progression in the cell cycle and they induce expression of p27 and p21 inhibitors of cell division [27]. Glucocorticoids can also elicit DNA fragmentation via caspase-independent pathways [28]. Another group of mechanisms of glucocorticoid mediated regulation of cell functions and survival are their interactions with nuclear factor kappa B (NF-κB), its binding site and its inhibitory IκB protein [29].

Also, weight loss, decreased appetite and low food intake can significantly supress immune response and this is a group of symptoms typical for major depression [30–32]. On the other hand those symptoms can also influence the level of exposure of gut-associated lymphoid tissue (GALT) to food antigens what further influences production of antibodies against food antigens [33].

On the other hand, a significant body of evidence indicates that depression is accompanied by activation of inflammatory response, increased levels of pro-inflammatory cytokines and their receptors, increased plasma concentration of acute phase reactants, increased parameters of oxidative and nitrosative stress and increased synthesis of detrimental tryptophan catabolites (TRYCATs) through the kynurenic pathway [34–37]. Additionally, recent promising factors have been proposed as practical biomarkers that could give a new perspective to understand the complex interactions between psychological stress, immunity and major depression. Evidence suggest that specific class of noncoding RNAs, called microRNAs (miRNAs) play a significant role in major depression and suicidal behavior what has been reviewed by Serafini et al. [38]. Various miRNAs are reported to be involved in the development, physiology and diseases of the central nervous system. Some of them are reported to influence immune response and oxidative stress leading to excitotoxicity [39, 40]. MiRNAs are also known to have a specific role in regulation of cytokine genes and expression of glucocorticoid receptors what may also have a great significance in pathophysiology of major depression and its immunity [41, 42].

Animal and human studies demonstrate that psychological stress may influence the mechanics of intestinal barrier integrity leading to its disruption and the translocation of enteric bacteria and food derived allergens. This can lead to subsequent activation of inflammatory response [2, 43–45]. Furthermore, psychological stress may lead to increased intestinal paracellular permeability followed by specific IgG response against soluble food antigens [46]. Increased intestinal permeability is related to food hypersensitivity and inflammatory cytokines are key factors of permeability modulation [47–50]. It is also believed that any event causing epithelial barrier defects may underlie food allergen sensitization [51]. Interestingly, the role of intestinal barrier disruption and IgG mediated immunity against food antigens have been described in psychiatric diseases such as schizophrenia, bipolar disorder and autism and it is becoming a topic of growing interest in psychiatry research [52–54]. Also, the role of increased intestinal permeability to enteric bacteria in major depression was demonstrated by Maes et al. [55]. The same authors in follow-up of the abovementioned study revealed that length and chronicity of major depression significantly influences level of intestinal permeability [56].

Since there is a high concentration of high affinity Fc receptors in the brain, particularly in the limbic system and microglia, activation of Fc receptors by immunoglobulins G can lead to production of pro-inflammatory cytokines and behavioral effects [57]. As a consequence depressive and hyperactive behaviors were induced in animal studies after intracerebroventricular injection of IgG [58].

Previously we suggested a hypothesis which described how increased IgG response against food antigens with subsequent activation of inflammatory response could play a part in pathogenesis of major depression [59]. Briefly, multiple factors such as psychological stress (mediated by corticoliberin - CRH), proinflammatory cytokines, oxidative and nitrosative stress, dysbiosis and a further large group of detrimental factors could cause weakening of tight junctions (TJ) of intestinal enterocytes. This could lead to increased intestinal permeability to enteric bacteria and food antigens. Excessive absorption of food antigens through weakened intestinal epithelium could lead to type III hypersensitivity with formation of IgG immune complexes. Those complexes could form locally (e.g. near the site of antigen entry) or peripherally (e.g. deposition in blood vessels walls, synovial membrane of joints, on the glomerular basement membrane of the kidney, on the choroid plexus of the brain etc.) and this process would be accompanied by activation of complement system [60]. Cumulation of immune complexes might cause development of chronic inflammation. As a consequence, proinflammatory cytokines, cortisol, nitrosative and oxidative stress could activate enzymes of the kynurenic pathway leading to decrease of plasma tryptophan concentration and increased synthesis of neurotoxic, detrimental to the central nervous system tryptophan catabolites (TRYCATs), through the kynurenic pathway. Moreover, increased levels of proinflammatory cytokines (e.g. IL-1β, IL-6, TNFα) could activate the hypothalamic–pituitary–adrenal axis (HPA) and increase expression of inactive form of glucocorticoid receptor GRβ in proportion to its active form GRα leading to glucocorticoid resistance. Furthermore, multiple immune mediated mechanisms (e.g. TRYCATs, pro-inflammatory cytokines, immunoglobulins, complement system) could negatively influence serotoninergic, noradrenergic and glutamatergic neurotransmission of the central nervous system [59].

Taking into consideration all of the above we found it valuable to assess IgG response against food antigens in depressed patients. We measured the serum concentration of IgG against 44 food antigens along with the concentration of pro-inflammatory cytokines such as tumor necrosis factor-α (TNF-α), interleukin 6 (IL-6), interleukin 1b (IL-1b) which are related to inflammatory hypothesis of depression and are also associated with modulation of intestinal permeability [50]. Since production of IgG antibodies depends also on frequency of exposure to food antigens, we assessed the frequency of consumption/exposure to investigated food antigens.

We also measured plasma cortisol concentration to assess HPA axis activity and possible immunomodulatory effect of cortisol on other immune parameters investigated by us.

In our preliminary research, we hypothesized two possible scenarios of immune responses:

1. immune suppression of IgG response against food antigens where possible hypercortisolemia could be a significant modulator of immune response,

or:

2) since production of IgG against food antigens is believed to be physiological when the intestinal barrier is intact, we hypothesized that possible dysregulation of its permeability related to psychological stress and depression could result in more pronounced translocation of food antigens through the intestinal barrier leading to increased concentration of IgG antibodies in depressed patients as a way of activation of low grade inflammatory response.

## Methods

### Subjects

Sixty-three subjects participated in the study - 34 depressed patients and 29 healthy volunteers. Patients with major depression diagnosis were admitted to the Outpatient Clinic of Stanislaw Deresz Psychiatry Hospital (Bialystok, Poland). Patients undergoing serotonin selective reuptake inhibitors (SSRI) monotherapy or drug free at the admission were classified according to the Diagnostic and Statistical Manual of Mental Disorders (DSM-IV-R) diagnostic criteria of major depression (Table 1). Subjects with inflammatory, oncological and autoimmune disorders, diabetes, patients previously diagnosed with psychiatric diseases other than depression, psychoactive substances abusers, patients with organic brain dysfunctions, and smokers were excluded from the study. Moreover, changes in routine blood biochemical parameters, Body Mass Index <18.5 kg/m^2^ and >30 kg/m^2^, treatment with antipsychotic drugs, mood stabilizers, antibiotics, anti-inflammatory medications, glucocorticosteroids were also criteria for exclusion from the study. All subjects gave written consent after the study protocol was explained. The study was approved by the local ethical committee.

**Table 1:** Depressive episode characteristics

Variable	Patients *n* = 34
Depression characteristics	
First episode	55.88% (*n* = 19)
Recurrent MDD	44.11% (*n* = 15)
Melancholic MDD	50% (*n* = 17)
Atypical MDD	11.76% (*n* = 4)
Suicidal thoughts	17.64% (*n* = 6)
Antidepressant treatment	
Yes	47.05% (*n* = 16)
No	52.94% (*n* = 18)
Antidepressant medication type	
Escitalopram	23.52% (*n* = 8)
Sertraline	11.76% (*n* = 4)
Paroxetine	11.76% (*n* = 4)
Weight and appetite characteristics	
Weight loss	55.88% (*n* = 19)
(last month)	M = 2.29, SD = 2.939
Weight loss	58.82% (*n* = 20)
(whole episode)	M = 3.74, SD = 4.926
Weight gain	11.76% (*n* = 4)
(last month)	M = 0.32, SD = 0.945
Weight gain	11.76% (*n* = 4)
(whole episode)	M = 0.79, SD = 2.384
Decreased appetite	58.82%, (*n* = 20)
Not decreased appetite	41.17%, (*n* = 14)

*MDD* Major depressive disorder

All results are expressed as mean, SD

*n* = number of participants

### Biochemical measurements

Fasting blood was collected between 8.00 a.m. and 9:00 a.m. for food specific IgG, pro-inflammatory cytokines and cortisol measurements. The time of blood collection was particularly important in the context of cortisol measurements because of its diurnal pattern of secretory activity with the highest levels in the morning hours [61].

Sera from 63 participants (34 patients and 29 controls) were examined for the concentration of total IgG against 44 selected food products (Table 3) using enzyme-linked immunosorbent assay (ELISA) ImuPro 50 test, according to the manufacturer’s recommendations (R-Biopharm, Darmstadt, Germany) [62, 63].

Plasma TNF-α, IL-6, IL-1b concentrations were measured with high sensitive ELISA commercial kits according to the manufacturer’s recommendations (R&D Systems: TNF-α catalog number HSTA00D, IL-6 catalog number HS600B, IL-1b catalog number HSLB00C).

Plasma cortisol concentration was measured with ELISA commercial kits according to the manufacturer’s recommendations (IBL International, Germany: catalog number RE52061).

### Dietary parameters measurements

To assess possible influence of exposure (food consumption) to food antigens on IgG concentration we measured exposure to 44 food products and 2 groups of food products such as dairy (cow milk, cow milk cheese and products based on cow sour milk) and grains with gluten (gluten, barley, oat, wheat, spelt and rye). This measurement was based on patients’ reports regarding frequency of consumption of those products and was constructed into a five point Likert-type scale described as follows:

How often do you eat this product? 0 = never; 1 = almost never (≤ 1 monthly); 2 = sometimes (2–4 times monthly); 3 = quite often (2–4 times weekly); 4 = very often (5 ≥ times weekly). We divided these results in three groups (quartiles) described as high (> Q3), low (< Q1) and medium (others) exposure.

Weight change measurements were based on patients’ interview regarding weight change (kilograms) during last month of current depressive episode and during whole current depressive episode.

Patients’ appetite evaluation was based on their report of having decreased appetite or not having decreased appetite.

### Psychometric measurements

Psychometric parameters measurements were assessed using the 17 items Hamilton Depression Rating (HAM-D 17) [64], the Symptom Checklist 90 (SCL-90) [65, 66] and the Perceived Stress scales (PSS-10) [67]. All of those scales are available in Polish language translations and adaptations, and are highly valued for their reliability, and diagnostic quality [68–73].

HAM-D 17 is the questionnaire rating severity of depression by assessing in 3 to 5 points scales parameters such as mood, feelings of guilt, suicide ideation, insomnia, agitation or retardation, anxiety, weight loss, and somatic symptoms.

The SCL-90 is a 90-item self-reported multidimensional questionnaire measuring wide range of psychopathological dimensions, such as somatization, obsessive–compulsive symptoms, interpersonal sensitivity, depression, anxiety, anger–hostility, phobic anxiety, paranoid ideation, psychoticism and global severity index which is the sum of scoring from all the scale’s dimensions. Each item is rated on a five-point scale, ranging from ‘not at all’ to ‘extremely’.

PSS-10 is a ten-item, self-reported questionnaire assessing the degree to which recent life situations are appraised as stressful. Respondents indicate on a five-point scale ranging from 0 (never) to 4 (very often) how often they have felt or thought in a certain way during the period of past month. All responses are then summed to measure the level of perceived stress.

### Statistical analysis

Statistical analysis of interaction between exposure to food antigens and IgG concentrations was based on a two-way analysis of variance (ANOVA) and a post hoc Bonferroni test. To verify hypotheses which met assumptions for parametric tests we used two-tailed Student’s *t-*test, otherwise we used nonparametric Mann-Whitney *U* test. To test correlations between variables we used *r* Pearson’s correlation coefficient. To analyze correlations of TNF-α with IgG and cortisol with psychometric parameters we used *chi squared* (*χ* ^*2*^) analysis. To measure the significance of the difference between analyzed correlation coefficients we performed a Fisher r-z transformation test.

## Results

There were no significant differences in the male/female ratio between depressed patients and normal controls. There were no significant differences in age between depressed patients and the control group (Table 2). There were no significant gender differences in any of the IgG against food antigens. There were no significant relationships between the age of the subjects and serum IgG. Patients had significantly higher scores (*p* < 0.001) compared to controls in all scales used for psychometric evaluation such as HAM-D 17, PSS-10 and all subscales of SCL-90 (somatization, obsessive–compulsive symptoms, interpersonal sensitivity, depression, anxiety, anger–hostility, phobic anxiety, paranoid ideation, psychoticism and global severity index). The type of depression: first depressive episode vs. recurrent depression, number of depressive episodes, antidepressant treatment (patients during SSRI treatment or drug free) did not influence measured biochemical parameters.

**Table 2:** Demographic characteristics of patients and controls

Variable	Patients *n* = 34	Controls *n* = 29	*t* ^*a*^	*p*
Age (years) mean, SEM	M = 42.26 SD = 10.28	M = 42.79 SD = 10.31	*t* = 0.205	0.83
BMI (kg/m^2^) mean, SEM	M = 24.74 SD = 3.15	M = 24.10 SD = 2.88	*t* = 0.834	0.40
Sex			*x* ^*2*^	*p*
Female *n*	25	21	*x* ^*2*^ = 0.1	0.921
Male *n*	9	8		

All results are expressed as mean, SD

*n* = number of participants

^a^Statistical analysis by *t*-test

*x* ^*2*^ Statistical analysis by McNemar test

*p* < 0.05

### IgG concentration in patients and controls

There were no significant differences in mean IgG concentrations against measured food antigens between patients and controls (Table 3).

**Table 3:** IgG concentrations against food products in 34 depressed patients and 29 controls [μg/ml]

Food products	Normal controls (*n* = 29)		Depressed patients (*n* = 34)		*t* ^*a*^	*p*
	M	SEM	M	SEM		
Grains with gluten	62.54	14.51	63.85	16.95	−0.06	0.954
Gluten	17.14	3.1	18.07	4.25	−0.17	0.864
Barley	10.8	4.52	6.66	1.61	0.92	0.364
Oat	10.83	3.8	6.77	1.99	0.99	0.329
Wheat	9.6	1.9	12.41	4.02	−0.60	0.553
Spelt	5.89	1.57	8.61	3.33	−0.70	0.487
Rye	8.28	1.93	11.33	3.85	−0.67	0.504
Dairy	51.33	11.46	33.83	8.20	1.27	0.21
Cow milk	25.66	5.74	17.77	4.46	1.1	0.276
Cow milk cheese	10.91	4.9	5.86	1.25	1.07	0.289
Products based on cow sour milk	14.75	3.37	10.19	3.02	1.01	0.318
Goat milk, cheese	6.05	1.22	8.14	4.34	−0.43	0.67
Sheep milk, cheese	6.84	1.28	9.4	4.69	−0.49	0.627
Pork	4.33	0.37	5.28	0.99	−0.84	0.406
Beef	3.88	0.46	3.93	1.13	−0.04	0.968
Chicken	3.92	0.34	5.65	0.87	−1.73	0.088
Broccoli	5.44	0.88	5.08	1.15	0.24	0.809
Red cabbage	3.76	0.6	4.54	1.09	−0.6	0.551
Carrot	4.27	0.65	4.11	0.85	0.15	0.885
Cucumber	4.31	0.77	4.58	1.21	−0.018	0.856
Sweet pepper	3.36	0.59	4.48	1.12	−0.84	0.405
Tomato	7.14	1.9	5.23	1.00	0.93	0.537
Celery	3.59	0.5	3.86	0.76	−0.28	0.78
Soybean	3.52	0.36	3.82	0.65	−0.39	0.697
Pineapple	8.04	1.72	7.37	1.2	0.33	0.75
Watermelon	4.08	0.79	5.06	1.87	−0.45	0.654
Raspberries	2.25	0.24	3.59	1	−1.21	0.23
Cherries	4.05	1.13	4.29	1.08	−0.15	0.88
Poppy seeds	3.23	0.49	2.83	0.47	0.57	0.57
Almond	3.16	0.85	2.43	0.36	0.83	0.41
Hazelnut	2.49	0.41	3.44	1.13	−0.74	0.462
Peanut	4.28	0.83	4.49	0.58	−0.21	0.834
Pistachio	4.80	1.53	4.55	0.82	0.115	0.883
Linseed	3.46	0.7	4.82	1.66	−0.71	0.48
Sunflower seed	4.54	1.24	4.70	0.66	−0.11	0.91
Horseradish	5.5	1.62	5.77	1.36	−0.13	0.897
Turmeric	6.45	1.02	6.15	0.86	0.23	0.82
Garlic	6.81	2.31	6.30	1.75	0.18	0.859
Mustard seed	2.39	0.5	2.40	0.43	−0.01	0.992
Cod	6.7	0.5	7.46	0.72	−0.84	0.407
Crab meat	5.87	0.92	5.91	0.93	−0.31	0.975
Chicken eggs	30.15	7.02	27.89	6.9	0.23	0.82
Oyster mushroom	2.47	0.31	3.47	0.89	−0.98	0.33
Honey	6.02	0.75	4.91	0.89	0.94	0.353
Coffee	3.59	0.56	10.43	4.66	−1.34	0.184
Yeast	11.96	3.79	8.33	1.43	0.95	0.347

All results are expressed in mean, SEM

^a^Statistical analysis by *t*-test

*p* < 0.05

### IgG concentrations and exposure to dairy products

**Fig. 1:**
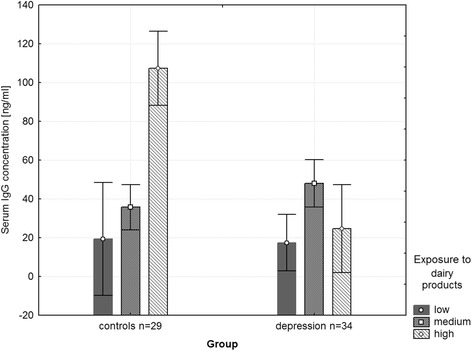
Interaction between IgG concentration [μg/ml] and exposure to dairy antigens in 34 depressed patients and 29 controls

We found significant interaction of IgG concentration against dairy antigens (combined data for all dairy antigens: cow milk, cow milk cheese and products based on cow sour milk) and exposure to dairy products between groups (F (2.63) = 3.92, *p* = 0.025, η^2^ = 0.12) and there was a significantly lower (post hoc *p* < 0.05) IgG concentration against dairy products in depressed patients (M = 24.72, SEM = 3.87) compared to controls (M = 107.35, SEM = 3.54) when there was a high exposure to dairy products (Fig. 1).

### Pro-inflammatory cytokines and cortisol analyses

There was significantly lower concentration of TNF-α in depressed patients compared to controls (Table 4). There were no differences in IL-6 and IL-1b concentrations between patients and controls. There was a significant difference in cortisol concentration between groups with higher concentration in depressed patients compared to controls (Table 4). Patients with melancholic depression had significantly higher (*M*_*rank*_ = 21.27) concentration of cortisol (*U* = 41, *p* = 0.006), when compared with the non-melancholic group of patients (*M*_*rank*_ = 12.16). We found 2 inverse correlations between cortisol concentration and food specific IgG concentration in patients’ group: peanuts (*r* = −0.37, *p* = 0.031), sunflower seed (*r* = −0.35, *p* = 0.043) and 4 correlations in the control group: broccoli (*r* = 0.38, *p* = 0.039), cherries (*r* = 0.5, *p* = 0.006), horseradish (*r* = 0.45, *p* = 0.014), honey (*r* = 0.49, *p* = 0.006).

**Table 4:** Cytokines and cortisol concentrations in patients and controls

Variable	Normal controls (*n* = 29)		Depressed patients (*n* = 34)		*t* ^*a*^	*p*
	M	SEM	M	SEM		
TNF-α [pg/ml]	1.7	0.13	1.09	0.4	4.256	<0.001**
IL-6 [pg/ml]	1.26	0.1	2.07	2.58	−1.662	0.101
IL-1b [pg/ml]	0.43	0.26	0.122	0.14	1.087	0.282
Cortisol [μg/ml]	136.35	10.29	174.76	12.08	2.375	0.021*

All results are expressed as mean, SEM

^a^Statistical analysis by *t-*test

**p < 0.01

*p < 0.05

### Cortisol correlations with psychometric parameters

We found significant positive correlations between cortisol concentration and the HAM-D 17 score in patients’ group (*r* = 0.442, *p* = 0.009) and inverse correlation between cortisol and HAM-D 17 in the control group (*r* = −0.373, *p* = 0.046). Cortisol also positively correlated with phobias in SCL-90 scale in the patients’ group (*r* = 0.531, *p =* 0.001). To measure the significance of the difference between above correlation coefficients we performed a Fisher r-z transformation test (Table 5).

**Table 5:** Pearson’s correlation between cortisol concentration and psychometric parameters

Variable	Normal controls (*n* = 29)		Depressed patients (*n* = 34)		Fisher r- z transformation	
	*r*	*p*	*r*	*p*	z	*p* ^*a*^
Cortisol	HAM-D 17					
	−0.373	0.046*	0.442	0.009**	−3.26	0.001**
	SCL-90 phobias					
	−0.083	0.668	0.531	0.001**	−2.54	0.011*

HAM-D 17: 17 items Hamilton depression rating scale; SCL90: Symptom Checklist 90

**p < 0.01

*p < 0.05

*p* ^a^ (two-tailed)

### TNF-α and IgG correlations

We found significant positive correlations between IgG concentration and TNF-α concentration. In the patients’ group IgG concentration of 38.63% (*n* = 17) food products significantly correlated with TNF-α concentration while such correlation was found only in 9.09% (*n* = 4) of food products in the control group. To measure the significance of the difference between the above correlation coefficients in TNF-α we performed a Fisher r-z transformation test (Table 6).

**Table 6:** Pearson’s correlation between TNF-α and food specific IgG concentrations

Food product	Normal controls (*n* = 29)		Depressed patients (*n* = 34)		Fisher r- z transformation	
	*r*	*p*	*r*	*p*	z	*p* ^*a*^
Broccoli	0.337	0.073	0.430	0.011*	−0.41	0.681
Red cabbage	0.204	0.288	0.441	0.009**	−1	0.317
Carrot	0.599	0.001**	0.414	0.015*	0.94	0.347
Cucumber	0.307	0.105	0.481	0.004**	−0.78	0.435
Sweet pepper	0.243	0.203	0.362	0.035*	−0.49	0.624
Tomato	0.781	0.001**	0.253	0.148	2.97	0.003**
Celery	0.358	0.056	0.373	0.030*	−0.07	0.944
Wheat	0.044	0.821	0.421	0.013*	−1.52	0.128
Spelt	0.093	0.63	0.431	0.011*	−1.38	0.167
Rye	−0.032	0.866	0.442	0.009**	−1.91	0.056
Cow milk cheese	0.085	0.659	0.415	0.015*	−1.34	0.180
Pork	−0.306	0.106	0.347	0.045*	−2.55	0.01*
Beef	−0.006	0.974	0.433	0.010*	−7.89	0.001**
Cherries	0.458	0.013*	0.304	0.081	0.68	0.496
Hazelnut	0.459	0.012*	0.030	0.866	1.75	0.080
Linseed	−0.143	0.458	0.464	0.006**	−2.43	0.015*
Horseradish	0.297	0.117	0.434	0.010*	−0.6	0.548
Mustard seed	0.366	0.05	0.419	0.014*	−0.24	0.810
Oyster mushroom	−0.130	0.5	0.417	0.014*	−2.16	0.03*
Yeast	−0.074	0.7	0.364	0.034*	−1.71	0.087

**p < 0.01

*p < 0.05

*p* ^a^ (two-tailed)

Values in *p* rows with significance in italic

### Length of depressive episode and IgG concentration

We found significant positive correlations of IgG concentrations and length of depressive episode (months) with IgG concentration against 11.36% of food products (*n* = 5): soy beans (*r* = 0.439, *p* = 0.009), oat (*r* = 0.639, *p* < 0.001), raspberries (*r* = 0.671, *p* < 0.001), honey (*r* = 0.474, *p* = 0.004) and coffee (*r* = 0.868, *p* < 0.001).

### Weight and appetite analysis

Most patients (55.88%, *n* = 19) reported weight loss (kg) in the last month of the current depressive episode (M = 2.29, SD = 2.939) and 20 patients (58.82%) reported weight loss during the whole current depressive episode (M = 3.74, SD = 4.926). Ten patients did not report any weight change (29.41%, *n* = 10). Four patients (11.76%, *n* = 4) reported weight gain in the last month of the current depressive episode (M = 0.32, SD = 0.945) and during the whole current depressive episode (M = 0.79, SD = 2.384). Most patients (58.82%, *n* = 20) reported decreased appetite. Fourteen patients (41.17%, *n* = 14) did not report decreased appetite. The control group did not report any changes in these parameters and for this reason we analyzed these variables in the patients’ group. Since in this study exclusion criteria were BMI < 18.5 kg/m^2^ and >30 kg/m^2^, none of the patients reached a degree of food deprivation and weight loss which could be classified as starvation (BMI < 16 kg/m^2^).

## Discussion

In this study we demonstrated immune suppression of IgG response to dairy proteins in depressed patients. This effect was statistically significant in groups with high exposure (consumption) to dairy products. We found significant correlations between exposure to dairy product and IgG concentration against dairy. Cytokines measurements revealed decreased concentration of TNF-α in depressed patients. There was hypercortisolemia in the patients’ group, more pronounced in patients with melancholic depression. Cortisol concentration was significantly correlated with depression severity measured with HAM-D 17 and cortisol concentration also significantly correlated with phobias in SCL-90. Moreover we found positive correlation between length of depressive episode and IgG concentration against 11.36% of food products.

Based on our results, we would suggest a possible causal link in which psychological stress (related to major depression) by activation of HPA axis leads to hypercortisolemia in depressed patients. High concentration of cortisol could directly suppress B lymphocytes function or could indirectly have the same effect through a decrease of TNF-α. Decrease of TNF-α could have immunomodulatory effects on B lymphocytes functions and production of IgG antibodies against dairy antigens.

TNF-α is a pleiotropic pro-inflammatory cytokine widely distributed in different types of immune cells and it regulates multiple functions of the immune system. One of its roles is the regulation of lymphocytes B functions. It is an autocrine grow factor for human B cells [74] and it is involved in the interaction with other T cell and monocyte derived cytokines. It also takes part in regulation of human B cell proliferation, immunoglobulins production [75] and augmentation of B cell responsiveness [76]. This cytokine plays a crucial role in humoral immune response and antibodies formation and as shown by Xu et al., TNF-α deficient mice have significantly impaired humoral response, especially production of IgG [77].

Cortisol is a well-known modulator and inhibitor of pro-inflammatory cytokines secretion. Hypercortisolemia, which we observed in our research in the patients’ group, could significantly influence TNF-α concentration. Moreover hypercortisolemia has a detrimental effect on B lymphocytes functions and antibodies production [21–24]. One of cortisol’s mechanism of action is inhibition of inflammation by abrogation of transcription factors such as nuclear factor-κB (NF-κB) and activator protein-1 which control production of pro-inflammatory cytokines [78]. Due to psychological stress and during depressive episodes the HPA axis is activated and higher levels of glucocorticoids are being produced. Glucocorticoids are responsible for the inhibition of proinflammatory cytokines e.g. TNF-α, IL-1b and IL-6 in the negative feedback mechanism [79]. However, with the time of exposure to stress and with the length of depressive episodes, glucocorticoid receptor resistance develops. This leads to lack of efficacy of the above mentioned negative feedback mechanism. As a consequence, despite of the high level of cortisol, levels of TNF-α, IL-1b and IL-6 increase what is a common characteristic of major depression. In our study we did not find increased level of proinflammatory cytokines but rather the opposite in the form of decreased TNF-α concentration. Glucocorticoid receptor resistance is not a sudden event and develops with the length of depressive episodes. Since 55.9% (*n =* 19) of our patients had the first episode of major depression, it is possible that they had not yet develop glucocorticoid receptor resistance to the degree which could lead to an increase of proinflammatory cytokines concentration. Also, our exclusion criteria were very strict regarding any possible inflammatory illnesses what could have additionally influenced the cytokines characteristics of our study group. All of the above indicate a modulatory role of hypercortisolemia along with decreased TNF-α concentration in the dampening of IgG response to dairy which we observed in our patients.

Another aspect which should be discussed is why dairy antigens were the ones where we found the most significant results compared with other food antigens. Results of our research are consistent with the paper by Volpi and Maccari [80]. Their research, performed on a group of almost 6900 healthy subjects, revealed that among the group of 160 food antigens, milk and its derivatives had one of the highest immune reactivity demonstrated by a high IgG response. Moreover, frequency of consumption of cow’s milk also corresponded with cow’s milk-specific IgG serum levels [81]. For this reason we believe that the IgG immune response against dairy could be more sensitive and reactive compared to the IgG immune response against other antigens evaluated in our study. This higher immune sensitivity and reactivity could be reflected in our research through dampened IgG immune response against dairy antigens.

In our research we did not find a significant difference in mean IgG concentration against food antigens between depressed patients and the control group. We believe that the following factors could have influenced our results. Moreover these characteristics could also contribute to previously described suppression of IgG response against dairy and decreased TNF-α production in depressed patients.

Well known symptoms of major depression are decreased appetite, lower food intake and weight loss. Those factors are also very significant in the context of immune modulation and response. Most of our patients 58.82% (*n* = 20) experienced loss of appetite and weight (kg) (M = 3.74, SD = 4.93) during the current depressive episode. It is possible that nutritional deprivation in our patients could have a significant role in suppression of their immune functions and IgG production. Animal and human studies indicate that nutritional deprivation suppresses immune response. This was documented in anorectic patients who demonstrated decreased peripheral IgG concentration compared to healthy controls [30]. Restriction of food intake can reduce memory of B cells, their immune response and IgG production. Those are believed to be mechanisms of energy preservation to the disadvantage of the immune response [31, 32]. Furthermore, decreased food consumption results in decreased presentation of food antigens to gut-associated lymphoid tissue (GALT) and the level of antigen-specific IgG depends on the dose of antigen [33]. Consequently, food restriction and elimination diet based on specific food IgG antibodies measurements revealed its therapeutic effect on neurological and gastroenterological symptoms in IBS, Crohn’s disease and migraine. This effect is believed to be related to decreased food specific IgG production due to decreased exposure to food antigens [63, 82–86].

Leptin which is a hormone regulating interactions between nutrition and immunity could also play a significant role in the observed results. Some of the vast immunomodulatory effects of leptin are regulation of functions of lymphocytes B, humoral response and secretion of TNF-α by lymphocytes B [87, 88]. Nutritional deprivation decreases the amount of circulating leptin and it is believed to be a peripheral signal of starvation leading to energy conservation when facing limited energy reserves. In the future further exploration of this field could be valuable [89, 90].

Previously, Maes et al. revealed that patients with chronic depression had higher immunoglobulin M (IgM) response against intestinal bacterial lipopolysaccharides (LPS) compared to patients with non-chronic depression, what suggested that the length and chronicity of depressive episode may influence intestinal permeability [56]. For this reason we performed analysis of correlations between IgG concentration against food antigens with the length of depressive episode. We found that IgG against 11.36% of food products (*n* = 5) positively correlated with the length of depressive episode (months). This result may suggest that with the length of the depressive episode lymphocyte B could be “regaining” their normal, initially decreased immune response with consequent increase of immunoglobulins G productions in later stages of depressive episodes.

Another possible scenario is that with the length of depressive episode we could expect gradual increase of intestinal permeability to bacterial and food antigens. This interpretation is consistent with the research by Maes et al., who revealed that length and chronicity of depression can significantly influence the intensity of inflammatory response and intestinal permeability [55, 56]. In chronic, recurrent depression we could expect increased concentration of pro-inflammatory cytokines, hypercortisolemia, increased CRH, increased nitrosative and oxidative stress, dysbiosis. All of those factors are known modulators of intestinal permeability that can weaken intestinal tight junctions leading to gut-derived inflammation. If so, it is highly possible that in chronic depression we could also expect increased intestinal permeability to food antigens and higher IgG response. In the above-mentioned research the criterion for chronicity of major depression was the duration of a depressive episode longer than two years. In our group of patients only one patient fulfilled the criteria of chronic depression. This could significantly influence the lack of difference in mean IgG concentrations between patients and controls. Furthermore, in chronic depression the appetite of patients could be not as disturbed as in the initial phase of the depression and they could be more exposed to food antigens.

Another important aspect is that we excluded from our research patients with inflammatory diseases including intestinal and autoimmunological disorders. It is possible that individuals with gastrointestinal and extra-intestinal autoimmune diseases who often suffer from comorbid depression could be a group of patients where increased intestinal permeability to food antigens could play a significant part in immune mediated pathogenesis of major depression. Previously, Fasano et al. proposed a hypothesis where increased intestinal permeability could be a source of antigens with subsequent molecular mimicry which could play a significant role in autoimmunity and further activation of inflammatory response [91].

For this reason focusing on patients with mostly first episodes of depression we see as a limitation of our study. Moreover due to the exploratory character of the research we analyzed a large amount of variables what in the context of multiple testing could be some limitation of the study. To minimalize this effect we performed analyses also in groups of food products such as dairy products or grains containing gluten.

Future research could consider verification of the hypothesis that in chronic, recurrent depression we could expect increased intestinal permeability to food antigens resulting with increased concentration of food specific IgG and subsequent amplification of the inflammatory pathways involved in major depression. Further research with larger group of patients with recurrent and chronic depression and measuring additional parameters e.g. leptin concentration, differences in IgG concentration between blood and cerebrospinal fluid would be valuable. Moreover, investigation of food specific IgG response in patients suffering from gastrointestinal and extra-intestinal autoimmune diseases and comorbid depression could be valuable.

## Conclusions

In this study we demonstrated immune suppression of IgG response against dairy proteins in patients with major depression. This effect was statistically significant in groups with high exposure to dairy products and exposure to dairy products significantly correlated with IgG concentration against dairy. We found decreased concentration of TNF-α and hypercortisolemia in depressed patients. Those results might play a significant role in suppression of IgG response. Nutritional deprivation could have been another factor which had a significant role in observed suppression of IgG response. There was no significant difference in mean IgG concentration against food antigens between patients and controls. It could be valuable to explore further a potential role of increased intestinal permeability to food antigens with subsequent IgG responses in patients with chronic, recurrent depression, and patients with gastrointestinal, and extra-intestinal autoimmune diseases with comorbid depression.

## Abbreviations

AIDS: Acquired Immune Deficiency Syndrome
BMI: Body Mass Index
CSF: cerebrospinal fluid
DSM-IV-R: Diagnostic and Statistical Manual of Mental Disorders
ELISA: Enzyme-linked immunosorbent assay
GALT: Gut-associated lymphoid tissue
HAM-D 17: 17 items Hamilton depression rating scale
HIV: Human Immunodeficiency Virus
HPA: Hypothalamic-pituitary-adrenal axis
IBD: Inflammatory bowel disorders
IBS: Irritable bowel syndrome
IgG: Immunoglobulin G
IL-1b: Interleukin 1 beta
IL-6: Interleukin 6
MDD: Major depressive disorder
PSS-10: Perceived Stress Scale
SCL90: Symptom Checklist 90
SSRI: Serotonin selective reuptake inhibitors
TNF-α: Tumor necrosis factor-alpha
